# Recommendations from a James Lind Alliance priority setting partnership - a qualitative interview study

**DOI:** 10.1186/s40900-020-00240-3

**Published:** 2020-11-19

**Authors:** Karin Jongsma, Juliette van Seventer, Anouk Verwoerd, Annemiek van Rensen

**Affiliations:** 1 Department of Medical Humanities, University Medical Center Utrecht, Julius Center for Health, Sciences and Primary Care, PO Box 85500, 3508 GA Utrecht, The Netherlands; 2 Department of Paediatric Rheumatology and Immunology, Wilhelmina Children’s Hospital, PO Box 85090, 3584 EA Utrecht, The Netherlands; 3 University Medical Center Utrecht, Utrecht University, Utrecht, Netherlands; 4 PGOsupport, PO Box 13040, 3507 LA Utrecht, The Netherlands

## Abstract

**Background:**

The James Lind Alliance (JLA) offers a method for research priority setting with patients, clinicians and carers. The method is increasingly used but publications primarily discuss the outcome of such projects, rather than reflecting on the JLA method itself. Scrutiny of the method is crucial in order to understand and correctly interpret its outcomes.

**Methods:**

We conducted a qualitative interview study with people involved in a JLA project into Juvenile Idiopathic Arthritis (JIA) (*n* = 30) to better understand the mechanisms, procedures and decisional processes during such a project and to formulate recommendations for those who consider starting a JLA project in the future.

**Results:**

Four main themes were identified: 1) motivations, goals and expectations 2) inclusivity, roles and representation 3) procedures and decision-making 4) outcomes and future steps.

**Conclusion:**

While the top 10 of ‘evidence uncertainties’ seems to take the centre stage in JLA projects, the ways in which these priorities are determined may be influenced by ‘process uncertainties’. We have formulated ten specific recommendations for future JLA projects. Reflection on and reporting of these process uncertainties would contribute to the improvement of JLA projects and increase the validity of the outcome of such projects.

## Plain English summary

The James Lind Alliance has developed an approach to identify shared research priorities of patients, health care professionals and carers. While the outcomes of such projects have often been described in the literature, the process itself is not. We have interviewed patients, health care professionals and parents of children with Juvenile Idiopathic Arthritis who participated in a research priority setting partnership of the James Lind Alliance. This interview study focused on their experiences with the process of establishing a shared research agenda. Our interviewees have pointed out 4 main topics that deserve attention in (future) agenda setting initiatives: 1) motivations, goals and expectations 2) inclusivity, roles and representation 3) procedures and decision-making 4) outcomes and future steps. We conclude that specific process details of a priority setting partnership are important because they affect the influence people have on the outcome. We have formulated 10 recommendations to further improve future priority setting partnerships that follow the approach of the James Lind Alliance.

## Introduction

Patient and public involvement (PPI) is increasingly fostered in health care research, clinical care and health care policy [1, 2]. PPI in research agenda setting is widely supported to tackle the research gap between research conducted by academic researchers and the needs that users such as patients, carers and clinicians have [3, 4]. As research agendas are supposed to direct future research paths, both clinical and societal relevance are at stake when the needs of end users are not properly addressed. The James Lind Alliance (JLA) offers a method for better aligning (bio)medical research agendas with the needs of patients, carers and clinicians by bringing them together in Priority Setting Partnerships (PSPs). By bringing together patients, carers and clinicians in a Steering Group, complemented with a widely distributed survey, so called evidence uncertainties can be inventorised and prioritised in a PSP. Although the outcomes of PSPs have been described in several academic papers, the approach itself and the ways in which decisions have been made is only rarely reflected upon. However, several factors in the process, including participant selection and the arrangement of decision-making processes, may influence the process and the outcome of such projects. One review has indicated the increasing use of the JLA approach for research priority setting and remarked that only little attention has been paid to disagreements and factors influencing the decision-making process in the reporting of these studies [5]. Another article, which evaluates a JLA PSP into Pressure Ulcers, has indicated that several decisions have to be made during the JLA project that are challenging and not straightforward. The reported challenges include the categorisation and dealing with different views and opinions [6]. However, that study does not draw on the experiences of PSP members themselves.

This study aims to fill these gaps by investigating the views, experiences and recommendations of participants involved in a JLA project into Juvenile Idiopathic Arthritis (JIA) through a qualitative interview study. We considered it particularly relevant to scrutinise the views of people involved in a JLA project, as their experiences allow us to identify challenges of the JLA method and to formulate recommendations for future PSPs. Correspondingly, the aim of this study was to shed light on the process of a JLA project by investigating the views and experiences of PSP members and the wider group of respondents of the survey. We believe this analysis will ultimately help to anticipate challenges and make thought-through decisions for those aiming to establish research priority setting projects in the future.

### About the JLA and JIA

The JLA is a non-profit organisation based in the United Kingdom that aims to bring patients, carers and clinicians together in the process of research priority setting. The values underpinning the JLA are 1) equal involvement, 2) balanced inclusion of patient, carer and clinician perspectives, 3) transparency of the process, methods, vested interests of those taking part in prioritisation and 4) a commitment to using and contributing to the evidence base [7].

The method developed by the JLA consists of several steps [7]. First, a Priority Setting Partnership (PSP) for creating a research agenda for a particular disease or condition is set up. This includes a steering group, consisting of balanced groups of patients and carers, and clinicians who have a decisive role throughout the process. A so-called ‘core group’ (usually the initiators/PSP leads, supported by other staff members such as the PSP coordinator and information specialist) selects participants for the steering group, manages the data collection and chairs the steering group sessions, and is supported by an advisor of the JLA. The second step involves collecting research questions from a wider group of patients, carers and clinicians through a survey. Thirdly, these questions are analysed by the core group with support of the steering group to determine whether these are in scope, and grouped into categories. Subsequently, questions are summarised into overarching questions by the steering group, after which an elaborate literature search is set up to verify that all summary questions are evidence uncertainties. The fourth step includes an online interim survey to a wider group of stakeholders, who are asked to prioritise the ten most important questions. This results in a shortlist of 20–25 questions, which constitutes the input for the final workshop. Participants of the final priority-setting workshop include an equal number of patients/carers, and clinicians and can include some steering group members. Then, the final top 10 is chosen.

This paper draws on the JLA project into Juvenile Idiopathic Arthritis (JIA), of which the study protocol has been published elsewhere [8]. JIA is the most common chronic rheumatic condition in childhood, with an incidence of 2–20 cases per 100,000 in high-income countries [9]. The JIA PSP was set up in The Netherlands in October 2018 by four patient, clinician and parent organisations. In this PSP, parents of JIA patients represent the group of carers. As JIA is a chronic condition that starts in childhood, therefore the JLA process has been adapted to include young children. Children were consulted via separate focus groups and their input was included and discussed in the steering group (see for further details [8]). The final top 10 has been published elsewhere (http://www.jla.nihr.ac.uk/priority-setting-partnerships/Juvenile-idiopathic-arthritis/top-10.htm) [10].

## Methods

We performed a qualitative interview study to investigate the views and experiences of patients, clinicians and carers who have contributed to the JLA PSP for JIA. Qualitative interviews are a valuable method to identify, better understand and juxtapose people’s experiences and views. The study is reported in accordance with the consolidated criteria for reporting qualitative studies (COREQ) [11].

### Participant selection and recruitment

Participants were recruited from two groups 1) members of the Steering Group (in the following: steering group members); 2) patients, carers and clinicians who filled out the survey to identify uncertainties and/or attended the final workshop (in the following: respondents). Members of the steering group were considered eligible when they had attended at least one face-to-face meeting of the PSP. Of the 18 members, 14 were available for an interview. Steering group members not available for interviews (*n* = 4) had various reasons: illness, a lack of time or they were not convinced their perspective was helpful, because they only participated in one meeting. All respondents were eligible, but only 43/303 were willing to be contacted for an interview. We selected and approached 34 (out of 303) potential participants based on their roles (patient, carers and clinicians) and geographic location, and informed them about the study via email. Sixteen were willing to participate in an interview (See Table 1). Others declined, because they considered their knowledge about the project too limited (*N* = 2), wanted to be compensated for participation (*N* = 1) or because of personal or logistical reasons (*N* = 4). Eleven participants did not reply to our invitation. Recruitment was ended when saturation was reached, i.e. when subsequent interviews no longer brought up new issues (‘coding saturation’) and the formulated themes were sufficiently understood (‘meaning saturation’) [12]. The initial survey did not inquire about age; therefore, prior to the interview, the participants were asked to confirm that they were at least 18 years of age. None of the participants dropped out of the study.

**Table 1:** Demographics of the Interviewees

Characteristic	Steering group members	Respondents
**Gender**		
*Male*	3	3
*Female*	11	13
**Role**		
*Patient*	4	5
*Carer*	2	6
*Clinician/expert*	6	5
*Core group*	2	0
**Geographical location**		
*North/east*	2	4
*Centre/west*	11	4
*South*	1	5

### Data collection

Semi-structured interviews were conducted by KJ (female trained qualitative researcher, PhD) and JS (female trained qualitative researcher). The topic list for the interviews was based on an analysis of the JLA guidebook and ethical and sociological literature about the JLA method, as well as discussions amongst the research team. The interviews consisted of open-ended questions related to experiences with participation in the project, roles and involvement, reflection on personal goals for participants and the goals of the JLA process in general, reflection on decisional process and outcomes, and possible ways of improving this process. The interviews with the steering group members included additional questions about the different steps of the process (See Supplemental File 1). The semi-structured design allowed participants to bring up or emphasise particular issues they considered relevant, whilst also ensuring some consistency in the topics that were discussed to explore how different respondents viewed these topics. Oral informed consent for participation in the interview, recording of the interview and data analysis of pseudonymised transcripts was obtained from all participants. The research ethics committee of the UMC Utrecht determined that this study was exempt from the Medical Research Involving Humans Act (research proposal no. 18/721). Interviews were conducted in Dutch and either took place at a location chosen by the participant (5/30), or by telephone (25/30). The interviews were audiotaped, transcribed verbatim, and pseudonymised.

### Data analysis

The pseudonymised transcripts were analysed thematically [13]. An initial coding list was developed based on the topic list, familiarisation with the data, and discussion in the research team. Subsequently, KJ and JS coded a sample of the transcripts individually, critically (re) read each other’s samples of coded transcripts, and the interpretations and suitability of the codes were discussed and compared amongst the research team. The coding list was evaluated and adapted, and all interviews were coded by KJ and JS using the academic software Nvivo 12. The meaning of individual text fragments was determined by interpreting them in the context of the whole interview with the participant in question [14]. In the course of analysis, codes were adapted, and additional codes were added to the coding list where necessary. A meaning pattern was identified across the data set, leading to the formulation of interpretative higher order themes. Throughout the process of analysis, the research team went back and forth between the different steps to allow for a constant comparison. In the last stage, relevant quotes were selected to illustrate the identified themes and translated to English.

## Results

### Demographics

A total of 30 semi-structured interviews were conducted in November 2018 (3 pilot interviews) and between February 2020 and March 2020 (*n* = 27). The interviews lasted between 12 and 63 min, with an average of 27 min. Interview participants were included based on different roles, including patients (Steering group *n* = 4; respondents *n* = 5), carers (Steering group *n* = 3; respondents *n* = 6), clinicians (Steering group *n* = 7; respondents *n* = 5). Two interviewees were core group members (those leading the project, chairing the steering group and managing the process), of which one clinician and one carer (in the results they are reported as steering group members to ensure the anonymity of their answers). At the time of the interview, 6 participants worked or lived in the north or east of the Netherlands, 15 in the centre of the Netherlands and 6 in the south. Table 1 shows the demographics of the interview participants. Some of the respondents (*n* = 1) and steering group members (*n* = 5) were present at the final workshop in which the top 10 of research priorities was made. Children and young adults were not interviewed for this study.

Four main themes were identified based on the experiences of the interviewees. When asked to reflect on the JLA approach, participants referred to 1) motivation, goals and expectations 2) inclusivity, roles and representation 3) procedures and decision-making 4) outcomes and future steps.

Table 2, 3, 4 and 5 list representative quotes that were selected to illustrate the identified themes (note that S refers to steering group, R to respondent; and _p refers to patient, _c to clinician and _o to parent). In the following, we indicate the participants’ role and characteristics only if it helps to contextualise their opinions in comparison to other participants.

**Table 2:** Quotes of interview participants that illustrate their experiences and perspectives on motivations, goals and expectations

	Theme 1: Motivation, Goals and Expectations
	**Motivation**
**1A**	“I’d consider the project successful when a top 10 is produced that is supported by all stakeholders. From the start that was the goal I had: developing a shared research agenda.” (S9_c)
**1B**	“And to hear from patients, you might think your research is interesting, but what I encounter in practice is this problem and I would really like a solution for it.” (R211_o)
**1C**	“Well, as I said before, my son has recovered, more or less without the use of medication, even though his condition was severe. And we have done certain things for that, and I want to stress that… surely, those aspects also have to be considered in research, because it is a completely different perspective and approach that can be successful.” (R184_o)
**1D**	“ In the West it is considered strange and when you have medication, it’s something special, and here nobody bats an eye, it is more inclusive. “ (R52_p)
	**Goals**
**1E**	“For me, from the patients perspective, it will be successful once we have a clear agenda or priorities of what should be investigated first in JIA research. And that in those priorities, the patients perspective is considered as well as parents and the clinicians. That it comes together into a shared research agenda, with the opinions of different stakeholders carrying equal weight. That would be a success.” (S11_p)
**1F**	Well yes, but what’s the value of a research agenda that is not implemented? Therefore I think that ideally the project would not stop after the formulation of the research agenda. Because we cannot be sure that academics will pick up that research agenda.” (S3_p)
	**Expectations**
**1G**	“ What I hear from these people from the UK is that the steering group, well facilitates a process, where people get to know each other better and learn about each others’ perspectives. It has been described before, that afterwards, people who were part of the steering group say that they have learned a lot from being part of the steering group.” (s1_o)
**1H**	“I had, well, the expectations were of course… Once we started I actually did not have any expectations, because it was the first time I participated in such a project. Also in my role as a parent in such a project, so I did not have high expectations. (S12_o)

### Theme 1: motivation, goals and expectations of the JLA project

#### Motivation

The majority of participants, especially clinicians, participated in the project because they deemed it important to jointly develop a research agenda (Quote 1A). Various (societal) groups may have different needs with regard to research and care than academic scholars, requiring better alignment of research with patients’ needs (Quote 1B). Other reasons for participation included helping others by sharing personal experiences (Quote 1C), raising awareness for JIA and ensuring representation of the specific regions in the Netherlands that were considered underrepresented in other patient involvement initiatives (Quote 1D).

#### Goals and expectations

Overall, there was great overlap between participants’ goals and expectations. Participants formulated as goal of the project to ‘develop a shared research agenda for JIA in which the perspectives of clinicians, patients and carers are equally represented’; and expected it to be feasible to reach that goal (Quote 1E). However, many participants stressed that the project should not end with the articulation of a top 10, as the ultimate goal is to influence research practice (1F). Furthermore, some steering group members anticipated an open process and expected participation in the steering group to be educational for all members (Quote 1G). Some participants indicated that they did not have expectations prior to the start of the project for a variety of reasons: because their involvement had been only marginal, they joined at a later stage, learned from other projects to keep their expectations low, or because it was their first time participating in such a project (Quote 1H). Some participants specifically hoped to see their individual perspective represented in the final research agenda.

### Theme 2: inclusivity, roles and representation

Oftentimes the inclusion of patients was mentioned as a crucial asset of the project. As JIA also affects young children, several steering group members reflected on the importance that young children are included (see quote 2A). Overall, people were happy with the selection of people in the steering group and felt respected in their respective roles (see quote 2B). A few clinicians and patients were critical about the extent to which the parents are representatives for adolescents, as adolescents and parents typically have diverging views (quote 2C). Academic researchers and funding agencies were argued to be missing in the steering group and by some argued to have additional value as the inclusion of these groups may contribute to a change of research practice. Others were critical of this inclusion, as they feared a conflict of interest when academics promote their own research.

Several interview participants reflected on the ways in which they could represent or speak for others. Some patients within the steering group stated that they continuously shifted between the role of (individual) patient with personal experiences and a role as representative of their patient organisation (See quote 2D). A similar role shift was noted by the parents (See quote 2E). Some physicians viewed their role as representative of a certain hospital, while others interpreted their role to represent a specific clinical profession. Furthermore, the importance of equal representation of all areas in the Netherlands was emphasised, as perspectives on disease and disability can differ between different regions.

With regard to their role within the steering group, some perceived a tension between being asked to participate as an expert, while simultaneously remaining receptive for the views of others. (See quote 2F). Some also mentioned that a few participants tried to voice their perspectives disproportionately strong, given the general willingness in this group to take all perspectives into account.

**Table 3:** Quotes of interview participants that illustrate their experiences and perspectives concerning inclusivity, roles and representation

	Theme 2: Inclusivity, roles and representation
	**Inclusivity**
**2A**	“And I think, you can’t only ask the adult, or young-adult patients how they have perceived it as a child. That is some sort of proxy, but the best thing is, to hear from the patients, the young patients themselves what they consider important. “ (S1_o)
**2B**	“Jee, would they take me seriously when I bring in my perspective? That feeling I lost already in the first half hour. I really think that they were listening with much respect for everyone. Regardless of whether you’re a parent or not. That is the benefit of voluntary participation; everyone is there because they chose to be part of this. It is not similar to a project you get assigned to at work, when people say: “ I’m not up for this”; you know? You see here that everyone has a positive attitude, but I also really felt respected.” (S12_o)
**2C**	“It is odd, but I think parents are different. They have their own view. Something that they consider important.. look, of course they can represent adolescents, but also not. Because they also see things differently sometimes.” (S7_p)
	**Roles & Representation**
**2D**	*“ Because I have noticed that it's better than solely your own opinion. You see, there is you and your own problems. But that is not what most of the people experience. Therefore I always tried to be transparent about what have I heard from others, also inside our organisation. To continuously provide feedback.” (S7_p)*
**2E**	*“I indeed participated as a parent, but I did not continuously think of my child this or that. Because there were several things, [..] several things we discussed that were not applicable [to my child], or did, but would only happen at a much later stage. I therefore have the feeling that I participated as a representative, even if you also should not think too much of it. But, well, you try to help everyone. (S12_o)*
**2F**	*“[..] patients and clinicians can see that very differently. And then in a discussion, that's complicated. Then you have to reflect and think to yourself, well, as a clinician you also shouldn't push your own perspective too much, you know?” (S6_c)*

### Theme 3: procedures and decision-making

Many participants praised the process of the JLA project, despite the fact that only few critically reviewed the methodology before deciding to join the project. Individual motivation was oftentimes based on positive expectations and association with the core-group and the JLA. Steering group members generally felt that they were provided the opportunity to bring in their perspectives in the group discussions and that they could express disagreement or settle disputes (See quote 3A). Also in the final meeting, which resulted in the top 10 of research priorities, the participants were provided sufficient opportunity to express their perspectives. The core group was praised for managing a balance between providing information and remaining open for feedback and disagreement, thereby enabling collective decision-making (see quote 3A). At the same time, some noted that the ways in which disagreements were dealt with were not clear to them (see quote 3B), while these decisions of the core group and organisation provided had significant influence on the project.

The interviewees varied in their views about how individual perspectives should be handled in a process of collective decision-making. Many considered it difficult to determine if and how such a plurality of views should be merged. Some argued the diversity of perspectives to be valuable in itself and did not see the necessity to always reach agreement. Especially in the context of JIA, which affects people in different phases of their lives, each with its own questions and challenges the diversity was stressed to be important. A few also considered it inevitable and even necessary that disputes emerged, in order to change the status-quo (see quote 3C). Others mentioned that disagreement should be resolved pragmatically, for example expertise-based or based on what is most efficient. Another suggestion was to give patients decisional authority in situations of dispute, to make up for not having been responsive to their voices previously or because they suffer from the disease (see quote 3D). Others argued that consensus should be sought for such group decisions, even if that takes a while. In order to achieve consensus, more formal decisional procedures such as democratic voting and majority decision-making may be required. Some interviewees outlined that democratic processes can only function well with a careful selection of representatives (3E). Other expressed a slight disappointment with unique perspectives getting lost in the process of democratic procedures aimed at consensus (see quote 3F).

Participants positively evaluated the clinicians involved in the project for having a friendly and open attitude and respecting all opinions equally in the steering group meetings. The steering group was praised for facilitating an open and pleasant atmosphere. Its members were furthermore respected and considered knowledgeable. Overall, people who are rhetorically strong were argued to have stronger influence on the decision-making process or outcome (see Quote 3G). In addition, a few participants expected existing hierarchies to affect the dynamics of the group, and considered it difficult to contest the views of clinicians (see quote 3G). A few blamed clinicians for pushing questions on the agenda that align with their professional interest and considered this to be a disturbance of the collective and open decision-making process. Some steering group members, especially patients, noticed hurdles to effectively and actively participate in phone meetings. Reasons for these hurdles included the nature of the topics discussed, the ways questions were phrased and unclarity about whom else was present in the call (see quote 3H). Overall, PSP members and respondents were satisfied with the balance between demandingness and results of the project. A few interviewees mentioned that the process of interpretation and understanding as part of the clustering of questions could have been improved (see quote 3I), by exploring them further in group discussions or focus groups, instead of a survey.

**Table 4:** Quotes of interview participants that illustrate their experiences and perspectives on the procedures and decision-making process

	Theme 3: Procedures and decision-making recesses
	**Disagreement**
**3A**	*“I never got the feeling that I couldn’t utter my disagreement or couldn’t comment on it. I found that very constructive of the whole process. It continued, without unnecessary delays leading nowhere. I think that both the personal and the interpretation of the perspectives of others played a role, but also that the process itself was helpful. For me it wasn’t a reason to hold back my comments or to ask questions for clarification.” (S7_p)*
**3B**	*“I’m not quite sure how they have dealt with everyone’s feedback, but I had the impression that it worked well.” (S10_c)*
	**Decision-making**
**3C**	*“Well, as soon as people work together, and everyone brings in their own knowledge and skills, that causes tensions. Sure. And that is what we need to come to what matters. And we need it to understand how differences play a role in practice and why. We need it to determine a new direction with each other. I mean, you cannot change anything without some tension. Indeed, with consensus nothing will ever change. And we started this to cause change.” (S3_p)*
**3D**	***“****Well, I think that questions of patients, indeed, should weight more and should be the central focus of that study.” (R141_c)*
**3E**	*“Look, you can, you cannot decide these processes by means of a majority, because, you know, then you have to be careful who is selected to be part of such sessions.” (R116_c)*
**3F**	*“The other side is that you would have hoped that very unique questions, that nobody had anticipated, would have been part of [the top 10]. And that's not the case now. These questions are, the way I read them, very aggregated questions that resonate with many people.” (R116_c).*
	**Hurdles and enablers for decision making**
**3G**	“Maybe the hierarchy was, you know, there are some people whom people are more responsive to or who can voice their own opinions stronger, who can express themselves well. Then the group follows easily.” (s11_p)
**3H**	“ Well, if I just consider the patients, then I think it’s been difficult for them to share anything in the phone meetings. That because, how should I say, the barrier is higher than in a face-to-face meeting. Before you bring in anything, you have to be certain that you say something meaningful. And I think the clinicians had less hurdles in such phone meetings.” (S7_p)
**3I**	“I think that if you ask for reasons why some people wanted to prioritise some of the questions, not in the larger sample, but in the top 10. Then you gain more insight in their worries and reasons and that they can explain what they consider important.” (R3_p)

### Theme 4: outcomes and future steps

Respondents and steering group members agreed that the process was more important than the actual outcome. Many reasoned that committing to such a process entails accepting the outcomes (See quote 4A). That this outcome was undecided until the end, was illustrated by a participant, who brought forward an example where a patient disagreed with the proposed top 10 in a subgroup at the final workshop - which resulted in a new top 10 (Quote 4B). This also indicates that responsiveness during the process is important for accepting the outcome. At the same time, participants argued that the overall outcome only differed marginally from their own preferences (Quote 4C) and that, since the process is well organised, the outcomes may be generalisable and representative for the whole group. Several interview participants considered the process extra successful when they recognised their own input or when their own question made it to the top 10 (Quote 4D).

Many participants argued that the outcome of the top 10 research priorities is valuable, while some were simultaneously critical, as none of the final priorities surprised them (Quote 4E). Some were not sure about whether the top 10 marked the end of the project or whether drafting research proposals is actually part of the JLA project. Many participants considered it a waste of money and time, should the project end with the publication of the top 10 priorities (Quote 4F). One steering group member suggested that aside from the top 10, important information has been gained in the verification phase: questions that were assessed as sufficiently answered and therefore were not considered evidence uncertainties, could be used to determine and decrease the gap between the availability of information in scientific literature and information available to patients. Participants almost unanimously considered impact on actual research practice to be important. Some argued that the translation of these questions into research proposals would suffice, while other considered the project successful once there are answers to their proposed questions (Quote 4G).

Interviewees proposed several steps to attain their desired outcomes. Many participants recommended drawing more attention of the public, academic researchers and funding agencies to the research agenda to increase the chance of impacting research practice. The publication on the website was argued to be insufficient for those purposes. Successful acquisition of research funding was considered crucial in order to influence actual research practice (see Quote 4H). Participants praised that the JLA for JIA project included a follow up process in which funders and researchers were involved after formulating the top 10. Many interview participants wanted to be included in future steps or at least be informed about the follow up of this project.

Furthermore, it was considered important to interpret or translate the research priorities into research questions (Quote 4I). To avoid the risk of interpreting the priorities differently than intended, it was argued that steering group members should be included to ensure accurate translation into research questions (Quote 4 J). Others preferred to include new people in this follow-up to bring in additional ideas. Some clinicians considered the continuous inclusion of patients and carers important, while others argued that it was solely up to them and academic researchers to formulate a research proposal and to determine the corresponding study-design. Moreover, it was considered unlikely that the complete top 10 will be successfully translated to research questions; therefore, a selection of the questions should be made. However, participants disagreed about the prioritisation of the questions: some argued for prioritisation according to the order of the top 10 (i.e. question 1 enjoying the highest priority) (Quote 4 K), while others argued that all 10 are equally important. A few patients and clinicians stated that the top 20 should be kept in mind, rather than solely the top 10.

**Table 5:** Quotes of interview participants that illustrate their experiences and perspectives on the outcome of the project and future steps

	Theme 4: Outcomes and next steps
	**Evaluation of the outcome**
**4A**	“*When you organise such a thing with a specific goal and you have a sound process, well yes, if you then, if you decide to participate then you support the process and, well i think, you then also have to accept what comes out of it.” (R114_o)*
**4B**	*“I remember, especially the last meeting, we had a top, we made a top 10 in one subgroup and then there was a girl who was a JIA patient. What do you think of it we asked and the whole top 10 was turned around. Nobody would [..] contest it, everyone accepted it. That was nice to see.” (S5_c)*
**4C**	*“Definitely no decision that I opposed, because then I would have said so. Some nuances I would have done differently. But if I have made the suggestion and it is not picked up, that’s absolutely fine. I don’t think it’s a problem to just go with it.” (S9_c)*
**4D**	*“I can’t deny that such a top 10 is important for research funding. In that sense, well yes at least that is what I think, I won’t deny that I solely participated out of interest, I also wanted to put forward my own research topics.” (S5_c)*
	**Future steps**
**4E**	*“I recognise the list, in my anamneses, I see regularly, the same sort of issues. I don’t think that the top 10 is shocking or a complete surprise, this is what you could have expected.” (R116_c)*
**4F**	*“It is a wonderful process, but if the process is to give people the impression that they have an influence and subsequently nothing is done with it, then I consider it pointless. A waste of time, because it costs much time and money, these sort of things.” (R114_o)*
**4G**	*“Look, we have to hope that people become enthusiastic and funding will be arranged, that questions will be picked up and there will be answers. Well, some of these you can’t really answer, but maybe a beginning that helps a bit.” (S8_o)*
**4H**	“*I think it is important that the future research will pick up the top 10, and that to funders we can say: hey listen this is a top-10 question and it’s important to fund this, because it’s what patients want. I hope that it can help in writing new funding applications and well that the top 10 is included when we are making new plans.” (s14_c)*
**4I**	*“That’s why I think it is important that well if our research agenda is finished, because the agenda does officially contain 10 questions, but they are not research questions. The James Lind Alliance refers to it as unanswered questions, but they are more like research topics. They are too broad for research. From each of those questions you can still formulate 10 different proposals that supplement each other.” (S1_o)*
**4J**	“If researchers would pick it up, you have solved a part, but not all. Because they can still interpret the question in their own way. While it was not the intended question, you know. Well, I think it is important that in the next steps that patients are similarly included.” (S7_p)
**4K**	*“ [..] my expectation is that it will be a large task to come from the question on place 1 to a sequence of good research, which also will provide the answer. Because that is what you want. Sooner or later you want to check the box behind question 1.” (R116_o)*

## Discussion

To our knowledge, this is the first empirical study reporting on the experiences of participants of a JLA project. Our analysis indicates that research priority setting with different stakeholders is not straightforward, even in a well-known and structured manner as the JLA describes. The empirical analysis of our material suggests that the selection of the steering group members, the goals they set and approaches for settling disagreements have influenced the inventory and prioritisation of evidence uncertainties and therefore the outcomes of this project. Respondents and steering group members had similar perspectives, even if steering group members often had more observations to substantiate their position, because of their more pronounced role in the project. In what follows, we will reflect on the implications and relevance of our empirical study for decision-making in JLA projects, relate these to the broader academic literature, and provide recommendations for future JLA projects. We will first discuss how our findings align with the leading principles of the JLA and second we will outline some particular aspects of the JLA PSP for JIA, which is the project that our interview participants were involved in. Based on our analysis of challenges of the JLA method and the instructive ideas of the steering group members, we have formulated ten recommendations that are referred to in the discussion and listed in Table 6 below.

### Principles of the JLA method

While the JLA approach has been increasingly applied to a wide array of illnesses and conditions in the past few years, most articles report the top 10 research priorities of the project rather than reflecting on the approach that was employed [5, 15]. The JLA approach draws on Nominal Group Technique (a decision-making method), which is aimed at reaching consensus between different stakeholders. In the following, we will relate our findings to the leading principles of the JLA: equal participation, transparency, inclusivity and a commitment to using and contributing to the evidence base [7].

#### Equal participation

The JLA guidebook repeatedly describes the importance of ensuring that the voices of patients and carers are equal to those of clinicians during meetings and workshops. Also in the literature on patient and public involvement, responsiveness to the needs and opinions of patients has been described as an important factor [16–18, 19]. If researchers are not responsive to them, participants lack the motivation for (continuing) participation. Our study indicates that most people felt that they were sufficiently included and that they felt free to discuss and object to any decisions being made, yet phone meetings were less suitable ways for participation of patients and carers. An inclusive environment may however not be sufficient to ensure equal influence nor equal decisional-authority in collective decision-making. In other words, even if everyone has had the same chance to voice their perspective, the *influence* of these voices varied. Our respondents suggested several aspects that affected the influence of participants on the process and outcome. First, it was mentioned that the hierarchy (and perceived credibility) between clinicians and patients as in the clinical context is not simply dissolved when put in a different setting or environment. At the same time, some respondents (mostly clinicians) argued to be extra responsive to the perspectives of patients, and thereby giving unequal weight to these perspectives. It seems that the core group plays an important role in setting appropriate procedural conditions for tipping the balance to more equal influence on the decisional outcome: this includes the selection of participants (see recommendation 1, below) and the arrangement of decisional process Second, perspectives that affirm what one already believes may have had a stronger influence on top 10 priorities, as the JLA process is strongly focused on finding consensus. Third, also people with strong rhetorical skills were argued to have had a greater influence on the priority setting process, due to their ability to clearly formulate and express their perspectives (see recommendation 2, below).

#### Inclusivity

This JLA for JIA project included focus groups to involve younger children in the process of research priority setting. Overall, people were enthusiastic about the explicit involvement of children and in the final workshop often looked at the top 10 of the children (which was established in a separately organised focus group), in order to benchmark their own wishes and perspectives. The JLA guidebook recommends excluding non-clinician researchers from the voting procedure in the priority setting phases of the project, but mentions that they could be included in a JLA project for example as members of the steering committee or as observers (not voting members) of the final workshop. Most respondents argued that impacting research practice is the overarching goal of this project (recommendation 3), which goes well beyond the more specific goal of JLA formulating a top 10 of evidence uncertainties. Our interviewees formulated several suggestions for how that could be attained, such as the inclusion of funding parties or academics from the beginning of the JLA project. Or, alternatively, adjusting the JLA approach by including an additional step in which the steering group members or other patients, carers and clinicians are involved to translate the questions into research projects. Following such an approach, the participation of also non-clinician research may be desirable. Earlier involvement of this group (for example as observer) may be beneficial for creating support in the follow up phase. Future projects should keep in mind that such follow-up projects requires significant coordination and management, and involvement of some of the PSP members may be recommendable to ensure aligned follow-up. The so-called ‘Dialogue Method’, an alternative to the JLA approach for shared research priority setting, includes such a programming as well as implementation step [18]. In these projects, all stakeholder groups (often including academic researchers) develop their own agenda’s and merge these into one multi-stakeholder agenda during the dialogue phase of the process.

Furthermore, some steering group members indicated they switched between individual and collective standpoints in group discussions, and did not always clarify from which perspective they were arguing. Although individual experiences with the illness may bring useful experiential insight and context, they may simultaneously be influenced by circumstantial factors that are not necessarily representative for a collective. Collective standpoints should be clarified in terms of how individual voices have been aggregated into collective stances [20]. Note that this is similarly important for patients, carers and clinicians (see recommendation 4 below). It is surprising that virtually none of the published academic papers about the JLA projects reflect on questions of representation, while this is an important topic for determining whether the project has been inclusive, biased or otherwise flawed and representative voices are essential for the overall legitimacy and validity of the outcome (See recommendation 5).

JLA projects include members of different groups, with various experiences and interests that could collide, conflict or otherwise results in disagreement. A review of several JLA projects, however, pointed out that none of the reviewed projects reported on disagreements in the process [5]. Our interviews showed that there are several ways in which disagreements can be settled (See recommendation 6, below). One study that evaluated a JLA PSP project indicated that the process of prioritisation produced a tension between producing overarching questions while preserving authentic and particular questions, and that some patients and carers felt unheard by the group [6]. Another study supported this observation, by reporting that that the top 10 questions are very broad [21]. Our study indicated a similar tension with regard to the prioritisation: several of the interviewees indicated that the questions in the top 10 were general or unsurprising and signalled that unique perspectives got lost in the process. The loss of unique perspectives seems to be a trade-off from drawing on consensus-finding for collective decision-making. Consensus finding and democratic procedures are in themselves insufficient to include marginalised or unique perspectives; substantive diversity will inevitably get lost in such processes (See recommendation 7, below). Authority-based settling of disagreement or democratic decision-making with the purpose of steering clear of endless discussions were stressed by our respondents, which seems to support some pragmatic way of deciding and settling disagreement. This is, however, square to the principle of inclusivity in decision-making. Authority based decisions and democratic voting will exclude less strong voices with substantively valid points or even reinforce marginalisation, especially when group discussion or disagreement are suppressed [22].

#### Transparency

Many participants described explicitly or implicitly that they trusted the process and the core group. Thereby, trust seems to be a crucial element in the willingness of steering group members and respondents to collaborate (See recommendation 8, below). This trust is remarkable as none of the participants critically reflected on steps or the full process of the JLA. For example, it was not clear to everyone how the core group dealt with disagreement and participants stressed that they took the method more or less for granted. Furthermore, it was stressed that the core group has an important role to play in the PSP and has influence on the outcome through their preparations for the meetings, the choices they make during these meetings and their selection of participants.

In a JLA project, decisions that influence the project’s outcome are continuously made (e.g. who is selected for the steering group, how survey data is translated to researchable questions, how to decide and how to prioritise). Our respondents particularly stressed the importance of the prioritisation phase. The ways in which questions can be clustered and aggregated in overarching questions vary from more interpretative to more literal (see recommendation 9, below). As this step is more analytical and interpretative in nature, more guidance on the goals and methods to ensure the quality of this step would be desirable to harmonise this within and between different PSPs (see recommendation 10, below). Some participants argued that in this phase some nuances or the intended meaning of the question may get lost, and more reflection and facilitation of this process, for example by applying qualitative methods, such as focus groups, may help to better understand the context and meaning of the question as intended by the asker. The process of interim priority setting is not or only marginally discussed in scientific articles [23]. Given the importance of this step in the entire process, this is a shortcoming in the current literature. The JLA provides clear guidance on determining whether questions are evidence uncertainties, but only provides marginal and a strongly quantitative approach to the prioritisation of the questions (see recommendation 8, below). Regular feedback loops may be useful as an internal validity check for process steps that involve translation or re-formulation or any form of aggregation or clustering of the ‘raw’ input. Feedback loops and on-going reflection on the process could also increase the transparency of the process.

Based on the results and the reflection on our results in the contexts of other studies and the broader academic literature, we have formulated the following recommendations that could help to navigate between the different leading values of the JLA method. Note that in the JLA for JIA, recommendations 1 and 3 have been followed up and have been praised by the participants in our study.

**Table 6:** Recommendations for JLA PSPs

	Recommendations
1	The core group in future JLA projects should not solely select steering group participants based on the knowledge they bring in, but should be conscious of their deliberative virtues and the influence on the dynamic of the group to ensure an inclusive PSP
2	Rhetorical skills of individual PSP members may influence to a large extent the outcome of a JLA process. The core group should therefore carefully balance these skills in the different stakeholder groups. Additional training in this respect may be offered to individual participants to ensure equal participation.
3	Participants expect that the JLA top 10 will be used to have impact on research practice. It is therefore recommended that core group members, already in an early stage, plan ahead for the implementation of the top 10.
4	Steering group members should clarify in meetings from which perspective they speak: from one’s own experience or as a representative of a group
5	Validated methods for interest clustering and aggregation of the individual questions into overarching questions during JLA projects should be more explicitly discussed in the academic literature.
6	The steering group should reflect and report on how they have dealt with disagreements and what mode of decision making they have applied to better interpret the outcome of JLA PSPs, by documenting dissenting votes or dissenting opinions.
7	The research community should be made aware of ‘background’ documentation of the top 10 research priorities, to draw attention to more unique perspectives and authentic questions and to make the translation of research priorities into research projects responsive to the intended questions.
8	Composition of the core group deserves careful deliberation. As participants base their decision whether or not to participate at least partly on the degree of trustworthiness of the core group, selection of its members is critical.
9	A JLA project generates more relevant information next to the Top 10. For example during the verification phase where extensive literature searches are performed. The core group should strive to make also this (supplemental) information available to the patient and research community
10	The JLA should provide more guidance to deal with ‘process uncertainties’, especially during the interim prioritisation phase

### JLA for JIA

A JLA project for JIA provided the basis for this study. While the observations and recommendations can be used in JLA projects for other conditions, some of the results may carry extra weight for conditions that share characteristics with JIA. JIA is a common chronic rheumatic condition in children, which can persist in adulthood and comprises a relatively small patient group. In the Netherlands, JIA patients up to 18 years old are treated by paediatric rheumatologists and afterwards by general rheumatologists. Some patients are also treated by an ophthalmologist or other clinicians. In other words, it is an illness that demands attention from several clinical disciplines. As children, adolescents and adults are affected, the questions, concerns and research priorities of these groups may vary. Children were for example more concerned with being able to play with their friends, whereas adolescents had questions about independent living and adults for example about therapies. Furthermore, there are different ‘forms’ of JIA, that vary in terms of severity of symptoms, meaning that needs and priorities will differ. The variety of clinicians, life stage and severity of the condition, entails that JIA comprises a varied patient group. It can be risky to bring these groups together in a PSP, as the expertise and needs may differ too much to formulate shared priorities. For JIA this worked out well, probably because the group of clinicians knew each other to some extent and are used to working interdisciplinary, all geographical locations have been included and steering group members had been selected based on their willingness to collaborate and deliberate with other stakeholders. Furthermore, in the JIA community there have been other patient involvement projects, which may have sensitised some steering group members for such collaboration. The inclusion of younger children in JLA projects is not standard, but was considered crucial for being truly inclusive. Children were involved in two focus groups, and their input was included in the steering group meetings and the final workshop (findings are published elsewhere). This project indicates that even if the JLA approach is best suited for a homogeneous, well-defined condition, also in a relatively heterogeneous and varied group a shared top 10 was formulated, which is supported by those who have been involved. Also for this project, however, the proof of the pudding remains in the eating: whether the ultimate goal of the participants, to change JIA research practice, will be attained remains to be seen.

### Limitations

Our qualitative interview study should be understood in the context of the following limitations. First, the scope of our study was relatively broad. As it was the first large and in-depth interview study on the views and experiences of respondents and steering group members of a JLA project, we chose to conduct an exploratory study to allow participants to bring up issues they considered relevant. Saturation was reached on the codes and identified themes, however, future research could explore these topics in more depth. Second, qualitative interview studies are prone to interviewer- and researcher bias; a different interviewer could have focused their attention on different aspects of the respondents’ answers, and different researchers could have selected or grouped the identified codes and themes differently. The analysis of our data has been done by two researchers, in order to improve the rigour of the analysis. Third, the participants have contributed to a JLA project focused on JIA, their experiences and this context may have affected the ways in which our participants perceive and evaluate the process and respective recommendations. Nevertheless, given that most interviewees reflected on the process in general and outlined where aspects had to do with particularities of the JIA context, we believe that the recommendations provided may help other groups in preparing JLA projects. Furthermore, the respondents of the survey were self-selected and possibly more than average motivated and open to discuss their own views and the project. This may limit the applicability of these results to all respondents and to respondents with different backgrounds.

It would be relevant to conduct research into the effects and effectiveness of the proposed recommendations that were formulated by our respondents, even if this is quite likely methodologically challenging. Additional qualitative studies of other JLA PSPs would be desirable to investigate whether the identified dynamics, challenges and structures that we have identified are also found in other JLA projects. Similarly, an ethnographic study to describe the process from an observant perspective may provide additional insights into the dynamics, structures and challenges of such projects.

## Conclusion

The participants in this project were particularly motivated to affect research practice, a goal that goes well beyond the scope of JLA projects in which top 10 research priorities are formulated. While the top 10 of evidence uncertainties seems to take the centre stage in JLA projects, the ways in which these priorities are determined and ways in which decisions are made, are seemingly influenced by ‘process uncertainties’. There is a strong relation between the way the priority setting process is organised and the influence people have on the outcome, and both deserve an equal amount of attention. More reflection on and evaluation of procedural aspects, disagreements and reflection on representativeness is recommended during JLA project and in reporting the top 10. This has several benefits: 1) It can help to clarify the navigation of the core group between ‘getting things done’ and being inclusive and transparent for the steering group members, 2) It can help respondents to interpret how is dealt with their questions and priorities, 3) It can contribute to the legitimacy of the outcome and 4) It could help to interpret the questions that have been prioritised in the top 10 for researchers and funders.

## Supplementary information

**Additional file 1.** Topic list Steering Group JLA PSP for JIA.

## Data Availability

The data that support the findings of this study are available on request from the corresponding author KJ. The data are not publicly available due to them containing information that could compromise research participant privacy.
