# Supplementary material for: Recommendations from a James Lind Alliance priority setting partnership - a qualitative interview study
Source: Res Involv Engagem. 2020 Nov 19;6:68. doi: 10.1186/s40900-020-00240-3 (PMC7678261; doi:10.1186/s40900-020-00240-3)
Supplement: Supplementary file 1 — Additional file 1. Topic list Steering Group JLA PSP for JIA. [file 40900_2020_240_MOESM1_ESM.docx]

**Topic list Steering Group JLA PSP for JIA**

1. Own experiences and involvement

- Experiences with the project – compare with expectations
- Involved from/representing which perspective

1. Reflections on the JLA PSP process and goals:

- Goals of this process
- Feasibility with this method
- Suitability of this method for this stakeholder
- Reflection on inclusiveness and representation of stakeholders

1. Reflections on the decisional process

- Influencing factors, both negative and positive
- Equal say of all stakeholders, both descriptive as normative evaluation
- Dealing with disagreement
- Other relevant factors for decision-making

1. Satisfaction

- With the decisions made
- With the outcome
- With your own role
- Suggestions for improvement

1. Future steps

- For the outcomes of this project
- Lessons learned
